# Bee (Hymenoptera: Apoidea) Diversity and Sampling Methodology in a Midwestern USA Deciduous Forest

**DOI:** 10.3390/insects8030081

**Published:** 2017-08-04

**Authors:** Kenneth W. McCravy, Jared D. Ruholl

**Affiliations:** 1 Department of Biological Sciences, Western Illinois University, 1 University Circle, Macomb, IL 61455, USA; 2 USDA-Farm Service Agency, RR3 Box 129A, Carrollton, IL 62016, USA; jared.ruholl@il.usda.gov

**Keywords:** pan traps, indicator species, malaise traps, species composition, species diversity, species richness, vane traps

## Abstract

Forests provide potentially important bee habitat, but little research has been done on forest bee diversity and the relative effectiveness of bee sampling methods in this environment. Bee diversity and sampling methodology were studied in an Illinois, USA upland oak-hickory forest using elevated and ground-level pan traps, malaise traps, and vane traps. 854 bees and 55 bee species were collected. Elevated pan traps collected the greatest number of bees (473), but ground-level pan traps collected greater species diversity (based on Simpson’s diversity index) than did elevated pan traps. Elevated and ground-level pan traps collected the greatest bee species richness, with 43 and 39 species, respectively. An estimated sample size increase of over 18-fold would be required to approach minimum asymptotic richness using ground-level pan traps. Among pan trap colors/elevations, elevated yellow pan traps collected the greatest number of bees (266) but the lowest diversity. Malaise traps were relatively ineffective, collecting only 17 bees. Vane traps collected relatively low species richness (14 species), and Chao1 and abundance coverage estimators suggested that minimum asymptotic species richness was approached for that method. Bee species composition differed significantly between elevated pan traps, ground-level pan traps, and vane traps. Indicator species were significantly associated with each of these trap types, as well as with particular pan trap colors/elevations. These results indicate that Midwestern deciduous forests provide important bee habitat, and that the performance of common bee sampling methods varies substantially in this environment.

## 1. Introduction

Bees are arguably the most important group of pollinating animals, but there is growing evidence of declining bee populations [1,2,3,4]. Habitat destruction appears to be playing an important role in these declines [2,5], particularly in the Midwestern USA, where fragmentation of native landscapes through cultivation and urbanization has been especially extensive [6]. For instance, there is evidence that at least half the species of Midwestern bumble bees (*Bombus* spp.) have undergone declines in the past century [7].

In the Midwestern USA, restored and protected grasslands provide important habitat for native bees, and several studies of bee diversity in this region have focused on these habitats (e.g., [5,8,9,10,11]). Research suggests that agricultural crops near forested areas show improved visitation rates and higher bee species richness [12,13], but published studies of bees associated with Midwestern forests are limited. Increased knowledge of bees of Midwestern forests is needed to assess the role of these habitats in maintaining bee diversity and promoting pollination of agricultural crops.

Assessments of bee diversity are often based on results of trapping surveys. Trap types that have been used in bee surveys include pan traps, malaise traps, and vane traps. Pan traps are a common method of sampling bees [11,14,15,16,17]. These are colored pans or bowls that are usually placed on the ground, or sometimes elevated [9,11], and filled with a liquid such as soapy water that traps and kills the insects. Malaise traps [18] are mesh-fabric flight interception traps, with the Townes-style trap being a commonly used type [19]. Malaise traps have proven effective in collecting bees and other Hymenoptera [11,20,21,22,23,24]. Vane traps consist of two cross vanes with a collection container underneath. Insects contact the vanes and fall into the collection container. Vane traps have also been shown to be effective in collecting bees, particularly bumble bees [11,25,26].

Reliable estimates of bee diversity and species richness are essential for effective bee conservation. The relative effectiveness of pan traps, malaise traps, and vane traps has been evaluated in a west-central Illinois restored tallgrass prairie [11], and a few such comparisons have been done in savanna and forest environments (e.g., [21,27,28]), but information on the relative effectiveness of these methods in forests of the Midwestern USA is lacking. The objectives of this study were to (1) document the species of bees in a Midwestern USA upland deciduous forest and (2) compare the performance of different bee sampling methods in this environment.

## 2. Materials and Methods

This study was conducted from 12 April to 24 September 2011 in an oak-hickory forest (40.3650° N, 91.4075° W) at Western Illinois University’s Alice L. Kibbe Field Station, near Warsaw in Hancock County, IL USA. The station includes ~90 ha, with an adjacent ~590 ha owned by the Illinois Department of Natural Resources. These two holdings contain a mosaic of habitat types, including upland oak-hickory forests, floodplain forests, early successional forests, oak barrens, hill prairies, and restored tallgrass prairies. The study site was a contiguous forest of over 200 ha. Dominant overstory tree species included white oak (*Quercus alba* L.), northern red oak (*Quercus rubra* L.), shagbark hickory [*Carya ovata* (P. Mill) K. Koch], and black cherry (*Prunus serotina* Ehrh.). Abundant understory vegetation included bramble (*Rubus* spp. L.), prickly ash (*Zanthoxylem americanum* Mill.), fragrant sumac (*Rhus aromatica* Ait.), pointedleaf ticktrefoil [*Desmodium glutinosum* (Muhl.) Wood], Virginia creeper [*Parthenocissus quinquefolia* (L.) Planch.], and sedges (*Carex* spp. L.). Common spring ephemerals included bloodroot (*Sanguinaria canadensis* L.), spring beauty (*Claytonia virginica* L.), Dutchman’s breeches [*Dicentra cucullaria* (L.) Bernh.], wild blue phlox (*Phlox divaricata* L.), blue-eyed mary (*Collinsia verna* Nutt.), and wild columbine (*Aqilegia canadensis* L.).

Four study plots were established, with plots spaced 100 m apart and 50 m from the forest-tallgrass prairie interface. Each plot was a transect containing 18 traps: two groups of three elevated pan traps (one each of blue, white, and yellow), two groups of three ground-level pan traps (one of each color), two pairs of vane traps (one blue and one yellow in each pair), one Townes-style malaise trap with white roof, and one Townes-style malaise trap with black roof. Pan traps were 355 mL colored plastic bowls obtained from Greenbrier International, Inc., Chesapeake, VA, USA. These bowls were 4.0 cm tall, with a 9.0 cm bottom diameter and 15.5 cm top diameter. Vane traps and malaise traps were obtained from SpringStar Inc., Woodinville, WA, USA and Sante Traps, Lexington, KY, USA, respectively. Elevated pan traps were positioned 1 m above ground level via attachment to the top of a 3.8 cm by 3.8 cm wooden post [11]. Within each transect, the groups of pan traps, pairs of vane traps, and each malaise trap were randomly located, with 5 m between each trap. Within each group of pan traps and pair of vane traps, colors were randomly located as well. Pan traps and collection containers of vane and malaise traps were filled with water containing a few drops of dishwashing detergent to break surface tension. Traps were operated on clear, warm, calm days on 12 April, 28–29 May, 9–10 July, 20–21 August, and 23–24 September. Traps were operated for only one day in April; a large sample size was collected on that day and we were concerned that further trapping might have a negative impact on bee numbers. On each day of operation, traps were set at 0900 and collected at 1800 h. Collected bees were pinned, labeled, and identified to species level using a reference collection and the Discover Life online identification key (www.discoverlife.org).

Abundance, Simpson’s diversity indices, and species richness of bees collected were calculated. Mean numbers of bees and mean species richness collected by the four sampling methods, and the six pan trap color/elevation combinations, were analyzed using 2-way analysis of variance with trap type and plot as factors. Assumptions of normality and equality of variances were evaluated using the Shapiro-Wilk test and Brown-Forsythe test, respectively, and these assumptions were met for each analysis (*p* > 0.05). Means were separated using the Holm-Sidak multiple comparison procedure. Analyses of variance were done using SigmaPlot 13.0. Simpson’s diversity indices were compared between trap types using *t-*tests [29]. Holm’s step-down procedure was used to adjust *p*-values for multiple comparisons [30]. This procedure compares the *n*th smallest *p*-value with 0.05/(# of comparisons + 1 − *n*).

Minimum asymptotic species richness was estimated using two species richness estimators: the abundance coverage estimator (ACE) and the Chao1 estimator. ACEs and Chao1 estimates were calculated for each of the four sampling methods, as well as each pan trap color/elevation, using EstimateS Version 9.1 [31] with 100 randomizations and an upper abundance limit for rare species of ten. Probabilities that an additional individual sampled would be a previously undetected species (*q*_0_), and estimates of the number of individuals required to achieve a given percentage of *S*_est_, were also calculated [32]. Malaise trap data were included in these analyses for completeness, but they should be interpreted with caution because of small sample size.

The multi-response permutation procedure (MRPP) was used to compare bee species composition (species and their relative abundances) among different sampling methods. MRPP provides an *A*-value as a measure of divergence of species composition of different groups. *A*-values less than 0.1 are common, and values greater than 0.3 are considered relatively high [33]. Paired MRPP comparisons were done among the main sampling methods, as well as among the different pan trap colors/elevations. Holm’s step-down procedure was used to adjust *p*-values for multiple comparisons [30]. Bee species that were strongly associated with a particular sampling method were identified using indicator species analysis, or ISA [34]. This analysis provides an indicator value, ranging from 0 to 100, that measures the extent to which a species is exclusive (never collected by any other sampling method) and faithful (always collected by a particular sampling method) to a particular sampling method. Malaise trap data were excluded from MRPP and ISA because of small sample size. These analyses were done using PC-Ord Version 4.25 software.

## 3. Results

We collected a total of 854 bees, representing 16 genera and 55 species (Table 1). The seven most abundant species collected in this study, comprising 68.6% of the total, were *Andrena imitatrix* Cresson (28.5%), *Nomada pygmaea* Cresson (7.5%), *Lasioglossum versatum* (Robertson) (7.5%), *Lasioglossum subviridatum* (Cockerell) (6.9%), *Andrena erigeniae* Robertson (6.6%), *Augochlorella aurata* (Smith) (6.0%), and *Augochlora pura* (Say) (5.7%). The greatest numbers of individuals and species were collected on the April sampling date (624 individuals and 43 species). May sampling produced 59 individuals and 16 species, July sampling produced 116 individuals and 22 species, August sampling produced 31 individuals and 11 species, and September sampling produced 24 individuals and 9 species.

There was a significant overall difference in the numbers of bees collected by the different sampling methods (*F* = 21.369; *df =* 3.9; *p* < 0.001), with elevated pan traps collecting the most, and malaise traps and vane traps collecting the least (Table 1). Simpson’s diversity collected by ground-level pan traps was significantly greater than that collected by elevated pan traps (*t* = 5.366; *df =* ∞; *p* < 0.00001). There was a significant overall difference in species richness collected by the different sampling methods (*F* = 28.910; *df =* 3.9; *p* < 0.001). Elevated and ground-level pan traps yielded significantly greater species richness than did malaise traps and vane traps (Table 1). There was no significant effect of plot and no significant interaction effect for either comparison (*p* > 0.05). ACE and Chao1 analyses produced a roughly 5 to 6-fold range in minimum asymptotic species richness among different sampling methods (Table 1). With the exception of ground-level pan traps and malaise traps, observed species richness exceeded 80% of estimated asymptotic species richness, and vane trap observed richness exceeded 90% of asymptotic richness. Estimated sample size increases of roughly 5- to 7-fold, respectively, would be required for ground-level pan trap and malaise trap collections to reach at least 90% of minimum asymptotic species richness. *q*_0_-values for individual sampling methods indicated that, with the exception of malaise traps, an additional individual collected would be highly unlikely (2.7% to 5.8% probability) to be a previously undetected species.

Among pan traps, there was a significant overall difference in numbers of bees collected based on color and elevation (*F* = 7.052; *df =* 5.15; *p* = 0.001), with yellow elevated pan traps collecting the most (Table 2). There was also a significant effect of plot (*F* = 3.982; *df =* 3.15; *p* = 0.029), but no interaction effect (*p* > 0.05). Simpson’s diversity collected by yellow elevated pan traps was significantly lower than that of each of the other pan trap colors/elevations (*p* < 0.00044, each comparison). Overall, species richness differed significantly among pan colors/elevations (*F* = 4.282; *df =* 5.15; *p* < 0.013), with yellow elevated pan traps collecting greater richness than did blue elevated pan traps (Table 2). There was also a significant effect of plot (*F* = 7.710; *df =* 3.15; *p* = 0.002), but no interaction effect (*p* > 0.05). ACE and Chao1 analyses indicated that minimum asymptotic species richness was relatively high for elevated yellow pan traps, and that, except for white elevated pan traps, substantial increases in sample size would be needed to reach at least 90% of minimum asymptotic richness. *q*_0_-values for individual pan trap colors/elevations indicated that an additional individual collected would have a likelihood of 4.5% to 13.2% of being a previously undetected species.

Among malaise traps and vane traps, white malaise traps collected 16 bees and 10 species, and black malaise traps collected one bee. Blue vane traps collected 51 individuals and 13 species, and yellow vane traps collected 19 individuals and 3 species.

For all trap types (excluding malaise traps), the overall MRPP *A*-value was 0.30 (*p* = 0.000018). All three paired comparisons produced significant differences in species composition between trap types: elevated pan traps vs. ground-level pan traps (*A* = 0.24; *p* = 0.0060), elevated pan traps vs. vane traps (*A* = 0.25; *p* = 0.0068), and ground-level pan traps vs. vane traps (*A* = 0.27; *p* = 0.0054). ISA revealed seven species that were significantly associated with a particular trap type: three with elevated pan traps, three with ground-level pan traps, and one with vane traps (Table 3). Thirteen of the 18 bumble bees (*Bombus* spp.) collected were in blue vane traps.

The overall MRPP *A*-value was 0.22 (*p* = 0.00000001) for pan trap color/elevation comparisons. No paired comparisons were significant. Based on ISA, six species were significantly associated with a particular pan trap color/elevation: two with elevated yellow pan traps, one with ground-level blue pan traps, two with ground-level white pan traps, and one with ground-level yellow pan traps (Table 4).

The MRPP *A*-value for comparison of blue vs. yellow vane traps was 0.35 (*p* = 0.006). ISA revealed one indicator species, *A*. *imitatrix*, which was significantly associated with yellow vane traps (Indicator Value = 89, *p* = 0.031).

## 4. Discussion

Compared to grasslands and cultivated environments, there have been few studies specifically focused on bees in North American deciduous forests, and we are aware of none that have quantitatively compared the commonly used bee sampling methods employed in this study. In a study using vane traps (with clear plastic vanes) in the canopy and near the ground, 71 species of bees were collected in mature bottomland hardwood forest in northeastern Georgia [28]. Canopy traps accounted for 95% of all bees collected, and they collected 57 species vs. 47 collected by traps near the ground. This suggests that canopy trapping may yield increased species richness in forest bee sampling.

Of the 55 species of bees collected in our study, 43 (78%) were collected during the one-day April sampling period, and 624 of the total of 854 individuals (73%) were collected at that time. This probably reflects the importance of spring forest ephemerals to early season bees and the scarcity of flowering plant resources after canopy closure. For instance, *A*. *eriginae* is strongly associated with spring beauty, *C*. *virginica* [35], *Andrena* spp. and *Lasioglossum* spp. commonly visit bloodroot, *S*. *canadensis* and *Bombus* spp. are common associates of Dutchman’s breeches, *D*. *cucullaria* [36]. A majority of temperate deciduous forest understory herbaceous plants bloom before canopy closure [35,37]. In addition to contributing to maintenance of bee diversity, preservation of forest habitats may help in supporting bee pollination of agricultural crops locally [12,13]. Populations of pollinating insects can fluctuate substantially [38], and support of high bee diversity may promote a compensatory effect that maintains pollination levels [39]. However, the early peak in forest bee abundance followed by lower numbers may not correspond to crop pollinating phenology. It is even possible that agricultural crops may help support bee populations that are important in forest pollination. Further research is needed to clarify forest pollinator phenology and its potential relationship to crop pollination.

ACE and Chao1 analyses suggested that substantially more species of bees remained to be collected. Among the four main sampling methods, only vane trap observed species richness approached asymptotic richness. This result, combined with the relatively low observed species richness, suggests that vane traps alone would be a poor choice for achieving a synoptic bee survey in this environment. However, vane traps, and in particular blue vane traps, were the most effective method of collecting bumble bees (*Bombus* spp.) in this study, and *Bombus bimaculatus* Cresson were significantly associated with vane traps based on ISA. In some studies [25,40] blue vane traps have collected relatively high numbers of bumble bees and other large-bodied bees, suggesting that excessive collection of these bees by blue vane traps is a concern. In our study, bumble bees did constitute a high proportion of blue vane trap collections (13 of 51 bees collected, or 25.5%), but these traps collected relatively low numbers of bees in general. However, blue vane traps were much more effective than yellow vane traps overall, as was the case in restored tallgrass prairie [11]. *Andrena imitatrix* was significantly associated with yellow vane traps as well as elevated yellow pan traps in the present study, suggesting that in forests this species can be effectively sampled with elevated traps incorporating the color yellow.

Elevated pan traps collected significantly more bees than did any other trap type, and both pan trap methods (elevated and ground-level) collected greater abundance and species richness than did either malaise traps or vane traps. Furthermore, pan trap effectiveness varied substantially depending on color and elevation, with significant variation in species composition and several indicator species associated with a particular color/elevation. These associations may reflect the behavior and plant associations of these bee species. For instance, *N*. *pygmaea* were collected primarily in yellow and white ground-level pan traps, perhaps reflecting the host searching behavior of this cleptoparasite of ground-nesting bees, and the frequently green/yellow or white coloration of flowers in the plant genera *Euonymus*, *Rhus*, and *Rubus* that they visit [41]. The oligolectic *A*. *eriginae* and *Andrena violae* Robertson are associated with spring beauty (*C*. *virginica*) and *Viola* spp., respectively [35,42], and these two bee species were significantly associated with ground-level white and blue pan traps, respectively. The generalist bee species *A*. *imitatrix* and *Andrena pruni* Robertson were both associated with elevated yellow pan traps. These results are consistent with those of a study of the bee community of vernal pools in California [14], which found that yellow pan traps attracted mostly generalist bees. Yellow traps have been shown to collect a relatively wide variety of phytophagous insects [43]. *Andrena imitatrix* and *A*. *pruni* plant hosts include shrubs and trees such as *Crataegus* sp., *Prunus* sp., and *Salix* sp. [44], suggesting that these bee species tend to fly relatively high and therefore are more likely to be collected in elevated traps.

In a study in nearby restored tallgrass prairie at Kibbe Field Station, elevated pan traps also collected greater numbers of bees than did ground-level pan traps [11]. However, in that study blue pan traps collected the greatest numbers of bees, whereas in the present study blue pan traps collected the fewest, suggesting that pan color effectiveness can vary locally in relation to habitat. Blue was also the least effective color in northeastern USA forest bee collections [45], but it was relatively effective in southeastern USA forests [27]. Large sample size increases would be required to approach minimum asymptotic species richness using pan traps. This is particularly true for ground-level pan traps; for this trap type, estimated fold increases of 4.8 and 18.4 would be required to achieve 90%, and 100% of species richness, respectively. Incorporation of search-and-net collecting using insect nets to target specific bee species could increase species yield while limiting destructive sampling. This method can be especially effective when efforts are focused on particular host plants to collect oligolectic bees [46].

Malaise traps in particular collected very few bees in this study, whereas in the previously mentioned study in nearby restored tallgrass prairie malaise traps collected by far the greatest bee abundance and species richness among the four trap types used [11]. This suggests that the effectiveness of malaise traps in collecting bees is also habitat-specific. Insects must move upward along the malaise trap surface to fall into the collection container, and this probably involves phototaxis. The reduced sunlight within the closed canopy forest sampled in this study may retard bee upward movement and result in fewer captures. Of the 17 bees collected by malaise traps, only 1 was collected in an all-black trap, further suggesting that lack of sunlight and phototaxis limits malaise trap bee collections. In a study of robber fly (Diptera: Asilidae) diversity and habitat associations in Kibbe Field Station deciduous forests and restored prairies, malaise traps were relatively effective in sampling of these insects in forests [47,48], suggesting habitat-associated differences in behavioral response of these two insect groups to malaise traps.

Our results documented 55 species of bees inhabiting the forests of Kibbe Field Station, with potentially 15 to 20 species or more still to be discovered. This represents a small proportion (probably less than 15%) of the bee species richness in Illinois, and represents lower richness than has generally been found in grassland environments. Further research on the bee fauna of Midwestern forests is needed to more fully document this bee diversity and the role forests play in bee conservation. We also provide further evidence that bee abundance and species composition can vary substantially among sampling methods. Pan traps were the most effective of the four sampling methods tested in this study (see Table 5 for summary of sampling results). This is encouraging because pan traps are the least expensive and most convenient trap type, particularly ground-level pan traps. Increased sampling effort using this latter method would be relatively inexpensive and would likely result in collection of increased species richness, although very large sample sizes would likely be required to approach minimum asymptotic species richness.

## 5. Conclusions

West-Central Illinois oak-hickory forest harbors a fairly diverse bee assemblage, but with lower species richness that that found in Midwestern grasslands. Performance of different methods of sampling forest bees varied greatly. Malaise traps were ineffective, and vane traps collected relatively low bee numbers and species richness. Pan traps were most effective, particularly elevated pan traps. However, ground-level pan traps also collected relatively high bee numbers and species richness and, considering cost and convenience, showed the most potential for greater yields with increased sampling effort.

## Figures and Tables

**Table 1:** Totals (*n*) and mean numbers of bees (with standard deviations), Simpson’s diversity indices (D), and totals (*S*_obs_) and mean bee species richness (with standard deviations) collected using four sampling methods, with abundance coverage estimators (ACE) and Chao1 estimates (*S*_est_, with standard deviations) of minimum asymptotic species richness, *n* required to achieve a specified percentage of *S*_est_, and probabilities of an additional individual being a previously undetected species (*q*_0_) in oak-hickory forest in Hancock County, IL, USA, April–September 2011. Means followed by different letters are significantly different (*p* < 0.05; Holm-Sidak multiple comparison procedure); Simpson’s diversity values followed by different letters are significantly different (*t-*tests; *p*-values for multiple comparisons adjusted using Holm’s step-down procedure). ACE, *S*_est_, *q*_0_ and *n* required to achieve a specified percentage of *S*_est_ for malaise traps should be interpreted with caution because of low sample size. EPT = elevated pan trap, GPT = ground-level pan trap, MT = malaise trap, VT = vane trap, A = 12 April, B = 28–29 May, C = 9–10 July, D = 20–21 August, E = 23–24 September.

Species	EPT	GPT	MT	VT	Total	Dates Collected
Family: Andrenidae						
Genus: *Andrena*						
*A*. *carlini* Cockerell	2	2	0	0	4	A
*A. cressonii* Robertson	4	4	0	0	8	A
*A. erigeniae* Robertson	2	54	0	0	56	A
*A. heraclei* Robertson	1	0	0	0	1	A
*A. hippotes* Robertson	4	0	0	0	4	A
*A. ilicis* Mitchell	4	1	0	0	5	AB
*A. imitatrix* Cresson	197	23	4	19	243	A
*A. macoupinensis* Robertson	0	3	0	0	3	A
*A. nasonii* Robertson	21	11	0	1	33	AB
*A. nivalis* Smith	0	1	0	0	1	A
*A. nubecula* Smith	1	0	0	0	1	E
*A. perplexa* Smith	14	4	0	0	18	A
*A. pruni* Robertson	3	0	0	0	3	A
*A. spiraeana* Robertson	1	0	0	0	1	C
*A. violae* Robertson	0	3	0	0	3	A
Family: Apidae						
Genus: *Apis*						
*A*. *mellifera* Linnaeus	1	0	0	1	2	E
Genus: *Bombus*						
*B*. *bimaculatus* Cresson	0	0	0	4	4	AC
*B*. *impatiens* Cresson	4	1	0	9	14	BCDE
Genus: *Ceratina*						
*C*. *calcarata* Robertson	6	8	1	3	18	ABCD
*C*. *dupla* Say	3	3	0	1	7	AC
*C*. *strenua* Smith	0	5	0	0	5	A
Genus: *Melissodes*						
*M*. *agilis* Cresson	2	0	0	0	2	AC
*M*. *bimaculata* (Lepeletier)	0	1	0	0	1	C
Genus: *Nomada*						
*N*. *armatella* Cockerell	1	0	0	0	1	A
*N*. *dentariae* (Robertson)	0	1	0	0	1	A
*N*. *denticulata* Robertson	0	1	0	0	1	A
*N*. *depressa* Cresson	1	1	0	0	2	A
*N*. *pygmaea* Cresson	11	49	4	0	64	AE
Family: Colletidae						
Genus: *Colletes*						
*C*. *inaequalis* Say	0	2	0	0	2	A
Genus: *Hylaeus*						
*H*. *affinis* (Smith)	2	1	0	0	3	BCD
Family: Halictidae						
Genus: *Agapostemon*						
*A*. *virescens* (Fabricius)	1	0	0	0	1	C
Genus: *Augochlora*						
*A*. *pura* (Say)	33	6	1	9	49	ABCDE
Genus: *Augochlorella*						
*A*. *aurata* (Smith)	30	14	1	6	51	ABC
Genus: *Augochloropsis*						
*A*. *metallica* (Fabricius)	7	0	1	0	8	ABDE
Genus: *Halictus*						
*H*. *rubicundus* (Christ)	1	1	0	0	2	AC
Genus: *Lasioglossum*						
*L*. *admirandum* Sandhouse	0	1	0	0	1	A
*L*. *birkmannii* (Crawford)	1	0	0	0	1	B
*L*. *bruneri* (Crawford)	2	1	0	0	3	BCD
*L*. *cattellae* (Ellis)	7	1	0	0	8	ABC
*L*. *coeruleum* (Robertson)	4	3	0	0	7	AC
*L*. *coriaceum* (Smith)	2	1	0	2	5	BD
*L*. *hitchensi* Gibbs	11	6	1	2	20	ACDE
*L*. *imitatum* (Smith)	1	2	0	0	3	A
*L*. *obscurum* (Robertson)	2	1	0	0	3	BD
*L*. *pectorale* (Smith)	6	0	0	0	6	AC
*L*. *sagax* (Sandhouse)	1	0	0	0	1	A
*L*. *smilacinae* (Robertson)	12	17	0	0	29	ACDE
*L*. *subviridatum* (Cockerell)	22	33	1	3	59	ABCE
*L*. *timothyi* Gibbs	6	1	0	0	7	ABC
*L*. *truncatum* (Robertson)	1	0	0	0	1	A
*L*. *versatum* (Robertson)	35	19	2	8	64	ABCD
Family: Megachilidae						
Genus: *Megachile*						
*M*. *latimanus* Say	0	1	0	0	1	C
Genus: *Osmia*						
*O*. *bucephala* Cresson	2	0	0	0	2	A
*O*. *lignaria* Say	0	1	0	0	1	A
*O*. *pumila* Cresson	1	6	1	2	10	A
*n*	473	294	17	70	854	
Mean *n* per plot (SD)	118.25a (50.97)	73.50b (16.62)	4.25c (2.75)	17.50c (6.40)	213.50 (73.93)	
*D*	0.804a	0.905b	0.851ab	0.863ab	0.887	
*S*_obs_	43	39	10	14	55	
Mean *S*_obs_ per plot (SD)	22.75a (6.95)	19.25a (2.75)	3.00b (1.63)	7.75b (1.50)	32.50 (5.26)	
ACE	51.64	72.88	19.88	14.73	69.98	
*S*_est_ (SD)	53.54 (7.65)	87.00 (36.85)	33.05 (29.30)	15.47 (2.26)	73.72 (13.06)	
*n* required to achieve 100% of *S*_est_	2339	5701	355	162	6681	
*n* required to achieve 90% of *S*_est_	734	1718	134	---	1850	
*n* required to achieve 80% of *S*_est_	---	1141	92	---	1110	
*q*_0_	0.027	0.058	0.412	0.043	0.018	

**Table 2:** Totals (*n*) and mean numbers of bees (with standard deviations), Simpson’s diversity indices (D), and totals (*S*_obs_) and mean bee species richness (with standard deviations) collected using six pan trap color/elevation combinations, with abundance coverage estimators (ACE) and Chao1 estimates (*S*_est_, with standard deviations) of minimum asymptotic species richness, *n* required to achieve a specified percentage of *S*_est_, and probabilities of an additional individual being a previously undetected species (*q*_0_) in oak-hickory forest in Hancock County, IL, USA, April–September 2011. Means followed by different letters are significantly different (*p* < 0.05, Holm-Sidak multiple comparison procedure); Simpson’s diversity values followed by different letters are significantly different (*t-*tests; *p*-values for multiple comparisons adjusted using Holm’s step-down procedure). EPT-B = elevated blue pan trap, EPT-W = elevated white pan trap, EPT-Y = elevated yellow pan trap, GPT-B = ground-level blue pan trap, GPT-W = ground-level white pan trap, GPT-Y = ground-level yellow pan trap.

	EPT-B	EPT-W	EPT-Y	GPT-B	GPT-W	GPT-Y
*n*	73	134	266	76	130	88
Mean *n* per plot (SD)	18.25a (6.55)	33.50a (18.36)	66.50b (31.30)	19.00a (6.38)	32.50a (9.98)	22.00a (13.49)
*D*	0.883a	0.853a	0.670b	0.892a	0.822a	0.851a
*S*_obs_	18	25	33	21	26	21
Mean *S*_obs_ per plot (SD)	8.75a (3.69)	13.50ab (3.87)	15.50b (5.69)	9.00ab (2.83)	12.00ab (4.40)	9.25ab (2.06)
ACE	20.95	26.48	42.39	32.10	32.37	29.89
*S*_est_ (SD)	22.43 (4.74)	26.98 (2.24)	44.95 (9.13)	37.44 (14.65)	34.26 (6.85)	34.34 (12.32)
*n* required to achieve 100% of *S*_est_	283	278	1586	745	625	753
*n* required to achieve 90% of *S*_est_	111	---	527	264	226	268
*n* required to achieve 80% of *S*_est_	---	---	343	177	151	177
*q*_0_	0.082	0.045	0.045	0.132	0.077	0.102

**Table 3:** Indicator values for species significantly associated with a particular trap type based on indicator species analysis of bees collected in oak-hickory forest in Hancock County, IL, USA, April–September 2011.

		Indicator Values		
Species	*p*-Value	Elevated Pan Traps	Ground-Level Pan Traps	Vane Traps
*Andrena erigeniae*	0.006	2	96	0
*Andrena imitatrix*	0.012	82	10	8
*Andrena perplexa*	0.034	78	17	0
*Andrena violae*	0.046	0	75	0
*Augochlora pura*	0.034	69	13	14
*Bombus bimaculatus*	0.049	0	0	75
*Nomada pygmaea*	0.006	14	82	0

**Table 4:** Indicator values for species significantly associated with a particular pan trap color/elevation based on indicator species analysis of bees collected in oak-hickory forest in Hancock County, IL, USA, April–September 2011. EPT-B = blue elevated pan trap, EPT-W = white elevated pan trap, EPT-Y = yellow elevated pan trap, GPT-B = blue ground-level pan trap, GPT-W = white ground-level pan trap, GPT-Y = yellow ground-level pan trap.

		Indicator Values					
Species	*p*-Value	EPT-B	EPT-W	EPT-Y	GPT-B	GPT-W	GPT-Y
*Andrena erigeniae*	0.001	0	2	0	3	86	4
*Andrena imitatrix*	0.002	0	20	68	0	1	7
*Andrena pruni*	0.009	0	0	75	0	0	0
*Andrena violae*	0.015	0	0	0	75	0	0
*Ceratina strenua*	0.012	0	0	0	0	75	0
*Nomada pygmaea*	0.045	0	1	11	0	35	45

**Table 5:** Summary of number of bees and species richness collected using four sampling methods in oak-hickory forest in Hancock County, IL, USA, April–September 2011. EPT = elevated pan trap, GPT = ground-level pan trap, MT = malaise trap, VT = vane trap.

	EPT	GPT	MT	VT	Total
Number of Bees Collected	473	294	17	70	854
No. of bees/trap/day	2.19	1.36	0.24	0.49	1.32
Species Richness	43	39	10	14	55
